# Real-World Kidney and Glycaemic Outcomes Following Semaglutide Initiation in Adults with Type 2 Diabetes and Mild Chronic Kidney Disease

**DOI:** 10.3390/jcm15145577

**Published:** 2026-07-16

**Authors:** Syed Arman Rabbani, Haea Amar Alkoud, Mohamed El-Tanani, Elmoutaz Azmi Omar Mahmoud, Esraa Elsayed Hassan Ali Mohamed, Ghaith Kasim Zabadi, Hania Muzaffar, Anass Qasem

**Affiliations:** 1 RAK College of Pharmacy, RAK Medical and Health Sciences University, Ras Al Khaimah P.O. Box 11172, United Arab Emirateselmoutaz.22903019@rakmhsu.ac.ae (E.A.O.M.); esraa.22903007@rakmhsu.ac.ae (E.E.H.A.M.);; 2 Nephrology Department, Ibrahim Bin Hamad Obaidullah Hospital, Ras Al Khaimah P.O. Box 66212, United Arab Emirates; 3 Nephrology Unit, Internal Medicine Department, Faculty of Medicine, Zagazig University, Zagazig 44519, Egypt

**Keywords:** semaglutide, diabetic kidney disease, albuminuria, retrospective cohort study, United Arab Emirates

## Abstract

**Background:** Real-world kidney and metabolic responses to semaglutide in type 2 diabetes (T2D) and chronic kidney disease (CKD) remain poorly characterised, particularly in Middle East and North Africa (MENA) region. **Methods:** We conducted a retrospective, single-centre, paired-cohort study at a secondary care hospital in UAE. Adults with T2D and predominantly mild CKD newly initiated on subcutaneous semaglutide were included. Single-arm design without comparator; findings describe biomarker trajectories and cannot establish causality. Primary outcomes were within-participant six-month changes in serum creatinine, estimated glomerular filtration rate (eGFR), and urinary albumin-to-creatinine ratio (uACR). **Results:** A total of 324 patients were analysed (mean age 55.3 ± 12.4 years; 66.0% female; BMI 36.1 ± 6.9 kg/m^2^; median HbA1c 8.3% [IQR 7.2–10.0]; eGFR 87.0 ± 25.8 mL/min/1.73 m^2^; KDIGO G1–G2 in 82.7%; A2 92.6%, A3 4.6%). At six months, BMI fell by 2.0 kg/m^2^ and HbA1c by a median 2.0%; 52.0% achieved ≥5% BMI reduction and 87.2% achieved ≥0.5% absolute HbA1c reduction. Serum creatinine decreased by 3.6 µmol/L and eGFR rose by 3.7 mL/min/1.73 m^2^; given concurrent weight loss, these changes likely reflect reduced creatinine generation rather than true filtration improvement. A ≥30% eGFR decline occurred in only 0.9%. Geometric mean uACR fell by 19.8%; among participants with baseline uACR ≥ 30 mg/mmol, 66.7% achieved ≥30% uACR reduction. KDIGO G-category improved in 17.3% and was stable in 76.8%; the A-category remained stable in 95.7%. **Conclusions:** In adults with T2D, mild CKD, and substantial obesity, semaglutide was associated with clinically meaningful improvements in weight, glycaemia, and albuminuria over six months, supporting it as part of a layered cardio-renal protective strategy in routine care.

## 1. Introduction

Diabetic kidney disease (DKD) remains one of the most consequential complications of type 2 diabetes (T2D). It affects 20–40% of patients and is the single leading cause of end-stage kidney disease (ESKD) globally [1,2,3]. The burden extends well beyond kidney failure. Patients with both T2D and chronic kidney disease (CKD) face a cardiovascular mortality risk four- to eight-fold higher than those with T2D alone, making effective kidney protection a critical therapeutic priority [4]. For decades, renin–angiotensin–aldosterone system (RAAS) blockade was the cornerstone of that protection. The RENAAL trial established that angiotensin receptor blockade meaningfully reduces kidney failure and slows estimated glomerular filtration rate (eGFR) decline in patients with T2D and nephropathy [5]. A decade later, sodium–glucose cotransporter-2 (SGLT2) inhibitors redefined the standard of care. Across CREDENCE, DAPA-CKD, EMPA-KIDNEY, and the EMPA-REG OUTCOME kidney sub-analysis, these agents consistently reduced kidney composite endpoints through mechanisms independent of RAAS principally by restoring tubuloglomerular feedback and reducing intraglomerular pressure [6,7,8,9]. Yet even on optimised foundational therapy, kidney composite event rates remain approximately 11–13 per 100 patient-years in trial active arms [6,7]. A significant proportion of patients, particularly those with concurrent obesity, suboptimal glycaemic control, and persistent albuminuria, continue to progress despite current best practice.

Glucagon-like peptide-1 receptor agonists (GLP-1RAs) have attracted growing interest as a complementary approach to kidney protection in T2D. The cardiovascular outcomes trials for liraglutide (LEADER), dulaglutide (REWIND), and albiglutide each demonstrated significant reductions in major adverse cardiovascular events, establishing the class as cardioprotective in high-risk T2D [10,11,12]. Alongside these cardiovascular signals, consistent reductions in albuminuria emerged across trials, a finding later confirmed in two large meta-analyses, both reporting approximately 22% relative risk reduction in kidney composite endpoints across the GLP-1RA class [13,14]. Semaglutide has accumulated the most rigorous kidney-specific evidence within this class. In SUSTAIN-6, it reduced new or worsening nephropathy by 46% versus placebo, driven predominantly by urinary albumin-to-creatinine ratio (uACR) reduction [15]. A post hoc pooled analysis of the SUSTAIN 1–7 programme reported uACR reductions of approximately 20–25% versus comparators, with eGFR trajectory preserved over time [16]. Subsequent analyses from the STEP programme and the SELECT cardiovascular outcomes trial showed that these kidney effects extend to populations with obesity irrespective of diabetes status [17,18]. The landmark FLOW trial then settled the question of hard kidney outcomes, where semaglutide, in patients with T2D and CKD, reduced the composite of sustained ≥50% eGFR decline, kidney failure, and kidney or cardiovascular death by 24% [19]. It also slowed the annual eGFR decline by 1.16 mL/min/1.73 m^2^ per year and sustained uACR reduction throughout the 3.3-year trial. On the strength of this evidence, the KDIGO 2022 Clinical Practice Guideline for Diabetes Management in CKD now recommends long-acting GLP-1RAs as part of layered cardio-renal protection in eligible patients [20].

Despite this robust trial evidence, several important gaps persist. The landmark trials enrolled highly selected and controlled populations [6,7,19]. In routine practice, many patients with T2D and CKD present earlier in the disease course with predominantly albuminuria-driven CKD, preserved eGFR, marked obesity, and poorly controlled glycaemia. This real-world phenotype is underrepresented in trial cohorts, and whether semaglutide produces meaningful kidney and metabolic biomarker changes in these patients is not well established. A further gap is methodological. Few real-world studies have applied the KDIGO Cause–GFR–Albuminuria (CGA) framework to report outcomes in a way that is clinically interpretable for nephrologists [21]. Finally, there is a geographic gap. The Middle East and North Africa (MENA) region carries one of the highest global burdens of T2D and DKD, yet it is almost entirely absent from the real-world literature on GLP-1RA kidney outcomes [3].

These gaps frame the central question of the present study, namely whether the kidney and metabolic biomarker responses to semaglutide observed in controlled trials are reproduced in everyday clinical practice. To address this, we conducted a retrospective paired cohort study of adults with T2D and CKD newly initiated on subcutaneous semaglutide in routine clinical care at a secondary care hospital in Ras Al Khaimah, United Arab Emirates. The primary objective was to quantify within-participant changes in serum creatinine, eGFR, and uACR at six months. Secondary objectives included changes in BMI and HbA1c, KDIGO GFR and albuminuria category transitions using the CGA framework, and pre-specified clinically meaningful responder proportions. The study is single-arm, without a parallel comparator, and is designed to characterise real-world biomarker trajectories following semaglutide initiation rather than to establish causal treatment effects. Findings should be interpreted as hypothesis-generating, providing a real-world complement to the controlled trial evidence base rather than an independent test of efficacy. We contextualise these findings against the established trial evidence base, with explicit attention to interpretive caveats inherent to short-term, uncontrolled biomarker data.

## 2. Materials and Methods

This was a retrospective, single-centre, observational cohort study conducted at Ibrahim Bin Hamad Obaidallah Hospital, Ras Al Khaimah, United Arab Emirates. The study was approved by the Ministry of Health and Prevention Research Ethics Committee—RAK Subcommittee (MOHAP/REC/2025/22-2025-UG-P)—and conducted in accordance with the Declaration of Helsinki. Individual informed consent was waived given the retrospective and anonymised nature of the data. The study is reported per the STROBE guidelines [22] (File S1).

Electronic medical records of eligible patients were accessed following ethical approval. Patients with T2D and CKD (defined per KDIGO 2022 criteria as uACR ≥ 3 mg/mmol and/or eGFR < 90 mL/min/1.73 m^2^ persisting for ≥3 months) who were newly initiated on subcutaneous semaglutide between June 2024 and December 2024 were screened for eligibility. Chronicity was confirmed from the established CKD diagnosis documented in the patient’s medical record, reflecting prior clinical assessment; where a formal diagnosis was not recorded, eligibility was verified from at least two biochemical measurements separated by ≥3 months in the available medical records prior to the index date. Data were extracted in June 2025, allowing assessment of outcomes after a minimum six-month follow-up period. No a priori sample-size calculation was performed; all consecutively eligible patients initiated on semaglutide during the study period were included, representing a census of the available population.

Patients were included if aged ≥18 years with baseline measurements available within four weeks of semaglutide initiation and at least one paired six-month renal outcome. Key exclusion criteria were eGFR < 15 mL/min/1.73 m^2^ or renal replacement therapy at baseline; prior GLP-1 receptor agonist use within 12 months; SGLT-2 inhibitor initiation within three months of enrollment; active malignancy or systemic inflammatory condition; and pregnancy.

Subcutaneous semaglutide was prescribed as part of routine clinical care following the standard dose-escalation protocol: 0.25 mg weekly for four weeks and 0.5 mg weekly thereafter, with uptitration to 1.0 mg at physician discretion based on the patient’s response. Background medications were documented during the course of the study; foundational therapies were generally stable throughout the observation period and continued at the treating physician’s discretion.

Baseline measurements were defined as values recorded within four weeks of semaglutide initiation. Follow-up measurements were extracted from records obtained five to seven months post-initiation. Primary outcomes were within-participant changes in serum creatinine (µmol/L), eGFR (mL/min/1.73 m^2^) and uACR (mg/mmol) at six months. Secondary outcomes included changes in BMI and HbA1c, KDIGO GFR and albuminuria category shifts. Furthermore, proportions achieving pre-defined clinically meaningful thresholds—BMI reduction ≥ 5%, HbA1c reduction ≥ 0.5% (absolute), eGFR decline ≥ 30%, and uACR reduction ≥ 30%—were also evaluated. eGFR was calculated using the CKD-EPI 2021 creatinine equation, which does not incorporate a race coefficient, in accordance with current international recommendations. Blood pressure, lipid parameters, and other biomarkers were not systematically available in the clinical records and could not be assessed.

Statistical analyses were performed using IBM SPSS Statistics for Windows, version 29.0 (IBM Corp., Armonk, NY, USA). Continuous variables are presented as mean ± SD or median [IQR] according to distributional normality, assessed by the Shapiro–Wilk test. Within-participant changes were assessed using the paired *t*-test for normally distributed outcomes (BMI, serum creatinine, eGFR) and the Wilcoxon signed-rank test for skewed outcomes (HbA1c, uACR). uACR was analysed on the natural log scale; the antilogarithm of the mean log change yields the geometric mean ratio (GMR), representing the proportional change in uACR. KDIGO GFR and albuminuria category shifts are reported descriptively. A sensitivity analysis was performed on participants with complete paired data across all outcomes. Pre-specified subgroup analyses were conducted in participants with baseline eGFR < 60 mL/min/1.73 m^2^ and baseline uACR ≥ 30 mg/mmol. Exploratory associations between baseline biomarker values and the magnitude of six-month change were assessed using Spearman’s rank correlation. These subgroup and correlation analyses were exploratory and hypothesis-generating. Missing data were handled using a complete-case approach for each outcome independently; participants contributed data to all analyses for which they had complete paired measurements. All tests were two-sided; *p* < 0.05 was considered statistically significant.

## 3. Results

### 3.1. Study Population and Baseline Characteristics

**Figure 1:**
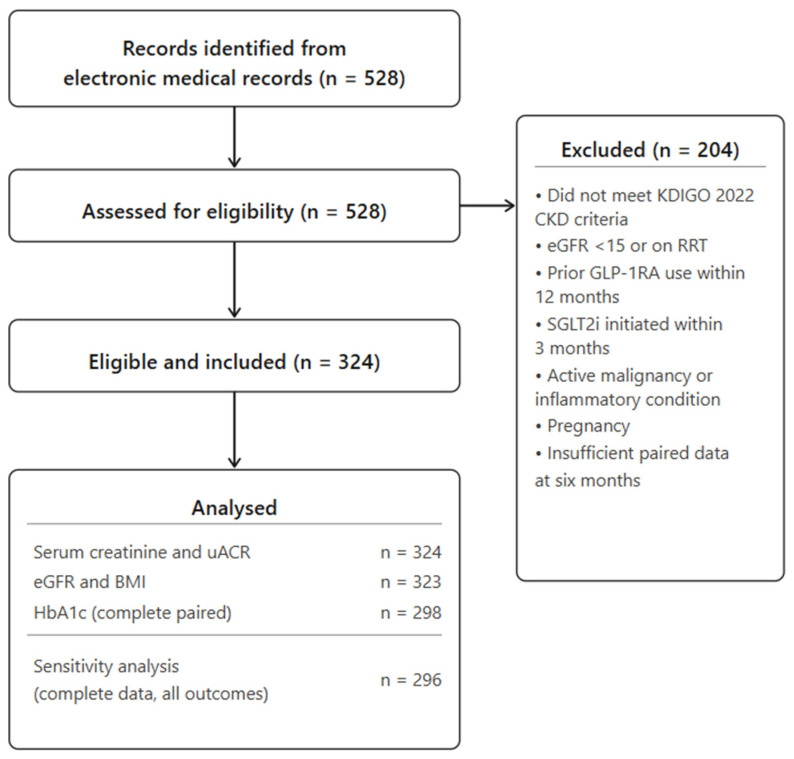
Participant flow diagram.

A total of 324 patients with type 2 diabetes and chronic kidney disease were included in the analysis (Figure 1). Paired baseline and 6-month measurements were available for serum creatinine (n = 324), uACR (n = 324), BMI (n = 323), and eGFR (n = 323); paired HbA1c was available for n = 298.

Baseline characteristics are presented in Table 1. Mean age was 55.3 ± 12.4 years and 214 (66.0%) were female. The cohort had marked obesity (BMI 36.1 ± 6.9 kg/m^2^). Baseline kidney function was generally preserved (eGFR 87.0 ± 25.8 mL/min/1.73 m^2^), with most participants in KDIGO G1–G2 (G1: 176/323 [54.5%], G2: 91/323 [28.2%]). Median baseline uACR was 7.7 [6.5, 9.9] mg/mmol. Using KDIGO albuminuria categories (A1 < 3, A2 3–30, A3 > 30 mg/mmol), baseline albuminuria was categorised as A1 in 9/324 (2.8%), A2 in 300/324 (92.6%), and A3 in 15/324 (4.6%).

### 3.2. Changes in Anthropometric and Glycaemia Outcomes at 6 Months

Within-participant changes from baseline to 6 months are summarised in Table 2. BMI decreased from 36.1 ± 6.9 to 34.1 ± 6.8 kg/m^2^ (mean change: −2.0, 95% CI: −2.2 to −1.8; *p* < 0.001). HbA1c improved with a median change of −2.0% (IQR: −3.3 to −1.1; *p* < 0.001), with substantial inter-individual variability.

### 3.3. Changes in Kidney Biomarkers at 6 Months

Renal biomarker changes are also shown in Table 2. Serum creatinine decreased from 80.5 ± 36.3 to 76.9 ± 32.7 µmol/L (mean change: −3.6, 95% CI: −5.4 to −1.8; *p* < 0.001), with a mathematically corresponding increase in eGFR from 87.0 ± 25.8 to 90.7 ± 24.7 mL/min/1.73 m^2^ (mean change: 3.7, 95% CI: 2.4 to 5.0; *p* < 0.001). This parallel pattern is consistent with semaglutide-associated weight and lean mass reduction lowering endogenous creatinine generation, which reduces serum creatinine and mathematically inflates the CKD-EPI eGFR estimate. These findings should not be interpreted as evidence of structural kidney function recovery. Albuminuria was analysed on the natural log scale; ln(uACR) decreased by −0.22 (95% CI: −0.28 to −0.16; *p* < 0.001), corresponding to a 19.8% reduction in geometric mean uACR (geometric mean ratio 0.80).

### 3.4. KDIGO Category Shifts

**Figure 2:**
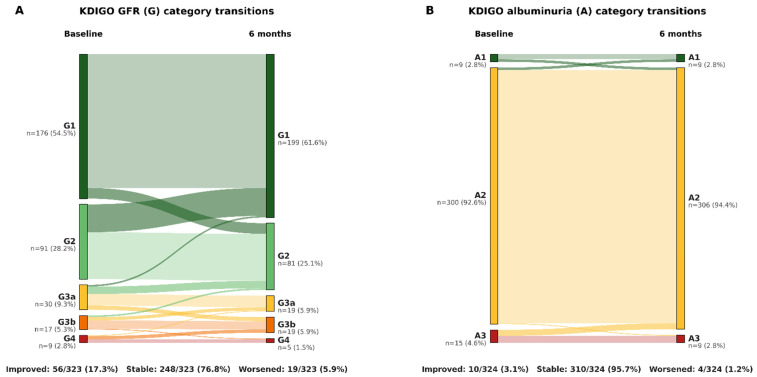
Patient-level transitions in KDIGO G and A stages from baseline to 6 months. Panel (**A**): GFR category transitions (n = 323). Panel (**B**): Albuminuria category transitions (n = 324). Flow widths are proportional to the number of patients transitioning between categories. Improved: movement to a lower-risk category; Stable: no category change; Worsened: movement to a higher-risk category.

KDIGO category shifts are shown in Table 3. Over 6 months, 56/323 (17.3%) improved by at least one GFR category, 248/323 (76.8%) remained stable, and 19/323 (5.9%) worsened. For albuminuria categories, 10/324 (3.1%) improved, 310/324 (95.7%) were stable, and 4/324 (1.2%) worsened (Figure 2).

Each ribbon represents a participant’s trajectory; ribbon thickness is proportional to the number of patients moving between categories. Diagonal flows indicate stable category; off-diagonal flows indicate category change.

### 3.5. Clinically Interpretable Endpoint Proportions

**Figure 3:**
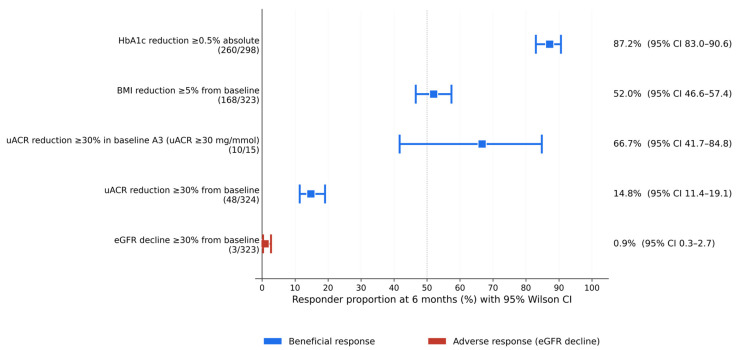
Clinically interpretable endpoints at 6 months.

Clinically interpretable endpoint proportions are summarised in Table 4. At 6 months, 168/323 (52.0%) achieved ≥5% BMI reduction, and 260/298 (87.2%) achieved an HbA1c reduction of ≥0.5% (absolute). A ≥30% decline in eGFR occurred in 3/323 (0.9%). Overall, 48/324 (14.8%) achieved ≥30% reduction in uACR; among participants with baseline uACR ≥ 30 mg/mmol (KDIGO A3), 10/15 (66.7%) achieved ≥30% reduction (Figure 3).

Squares denote point estimates of responder proportions; horizontal lines denote 95% Wilson score confidence intervals. Denominators reflect participants with paired baseline and 6-month values for the relevant outcome. The dotted vertical line marks 50% for visual reference only.

### 3.6. Sensitivity and Subgroup Analyses

Sensitivity analyses restricted to participants with complete data across all paired outcomes (n = 296) yielded findings consistent in direction and magnitude with the primary analyses, with persistent improvements in BMI, serum creatinine, eGFR, and ln(uACR) (all *p* < 0.001) and HbA1c (*p* < 0.001), supporting the robustness of the primary conclusions to missing data (Table S1). In the pre-specified subgroup of participants with baseline eGFR < 60 mL/min/1.73 m^2^ (n = 56), numerically larger improvements were observed in eGFR (mean change +6.7 mL/min/1.73 m^2^, 95% CI: +3.2 to +10.2; *p* < 0.001) and serum creatinine (mean change −11.6 µmol/L, 95% CI: −19.2 to −4.0; *p* = 0.003), accompanied by a reduction in ln(uACR) (mean change −0.35, 95% CI: −0.59 to −0.12; *p* < 0.001), consistent with greater absolute responsiveness in participants with more advanced kidney disease at baseline (Table S2). Exploratory Spearman correlation analysis across the full cohort demonstrated a significant inverse association between baseline uACR and Δln(uACR) (ρ = −0.540; *p* < 0.001; n = 324), confirming a continuous baseline-response gradient in which higher baseline albuminuria was associated with greater proportional reduction, and between baseline eGFR and ΔeGFR (ρ = −0.355; *p* < 0.001; n = 323), a pattern most likely reflecting the creatinine generation artefact and regression to the mean, rather than disproportionate filtration benefit in patients with lower baseline eGFR. In an exploratory analysis of the 15 participants with baseline uACR ≥ 30 mg/mmol (KDIGO A3), 10 (66.7%) achieved ≥30% uACR reduction (Δln[uACR] −1.41, 95% CI: −2.28 to −0.54; *p* < 0.001). Both subgroup analyses are exploratory, underpowered for formal inference, and susceptible to regression to the mean in the absence of a concurrent comparator; they should be considered hypothesis-generating only and interpreted alongside the primary cohort-level findings.

## 4. Discussion

### 4.1. Baseline Phenotype and Risk Profile

Our study population had marked obesity (baseline BMI 36.1 ± 6.9 kg/m^2^) and substantial dysglycaemia (baseline HbA1c 8.3 [7.2–10.0]%), consistent with a high cardio-renal–metabolic risk phenotype. Kidney status reflected predominantly preserved eGFR (G1–G2: 82.7%) with moderately increased albuminuria (median uACR 7.7 mg/mmol; KDIGO A2: 92.6%, A3: 4.6%). This pattern of albuminuria-driven CKD despite near-normal filtration is consistent with the KDIGO CGA risk framework, which defines CKD by either reduced GFR or persistent albuminuria and emphasises albuminuria as a strong independent prognostic marker for CKD progression and cardiovascular mortality across eGFR strata [20,21]. Accordingly, the findings of this study primarily apply to patients with early-stage, albuminuria-driven CKD and should not be extrapolated to those with advanced CKD (KDIGO G4–G5) or end-stage kidney disease, in whom the pharmacokinetic profile, tolerability, and kidney biomarker responses to semaglutide may differ substantially from those observed here.

### 4.2. Metabolic Effects: Weight and Glycaemic Control

A significant reduction in adiposity was observed at six months (BMI −2.0 kg/m^2^, 95% CI: −2.2 to −1.8), with 52.0% achieving ≥5% BMI reduction. This direction and magnitude are biologically plausible and align with semaglutide’s established weight-lowering efficacy across T2D and obesity trials [15,17,18]. Weight reduction is clinically relevant in CKD because obesity drives glomerular hyperfiltration and elevated intraglomerular pressure through haemodynamic and lipotoxic mechanisms—effects that can be attenuated by weight loss, as demonstrated in studies of bariatric surgery and intensive weight-loss programmes in obese populations [23]. Weight loss may therefore contribute indirectly to renal benefit through improvements in systemic haemodynamics, insulin resistance, and inflammatory tone [24]. In trials, semaglutide produced larger weight reductions with higher obesity doses (2.4 mg in SELECT/STEP) than standard diabetes doses [17,18]. The observed real-world BMI reduction is consistent with this pharmacological class effect [24].

HbA1c improved significantly (median change −2.0% [IQR −3.3 to −1.1]; mean −2.4%; *p* < 0.001), with 87.2% achieving ≥0.5% absolute HbA1c reduction. These findings are consistent with semaglutide’s glycaemic efficacy demonstrated in cardiovascular outcomes trials [15]. HbA1c lowering may attenuate glucotoxic injury to the kidney through inhibition of advanced glycation end-product formation, protein kinase C activation, and reduction in oxidative stress in the proximal tubule and podocyte; however, a meta-analysis of individual participant data from major glucose-lowering trials demonstrated that intensive glycaemic control provides modest but significant kidney protection, with much of the residual kidney disease progression attributed to non-glycaemic mechanisms [25]. Contemporary evidence further indicates that GLP-1RA’s kidney effects are not fully explained by glycaemic change alone, particularly for albuminuria endpoints [13,16,19,24]. The substantial inter-individual variability in HbA1c response likely reflects heterogeneous baseline control, background therapies, adherence, and disease duration, features typical of real-world cohorts [24].

### 4.3. Kidney Function Biomarkers

Serum creatinine decreased (−3.6 µmol/L, 95% CI: −5.4 to −1.8) and eGFR increased (3.7 mL/min/1.73 m^2^, 95% CI: 2.4 to 5.0), with ≥30% eGFR decline occurring in only 0.9% of participants. These findings require conservative interpretation. Creatinine is a product of muscle creatine metabolism; semaglutide-associated weight and lean mass reduction decreases endogenous creatinine generation, causing serum creatinine to fall and the CKD-EPI equation to yield a higher eGFR estimate independently of any genuine change in glomerular filtration rate [24]. This creatinine artefact represents a well-recognised confound in observational GLP-1RA kidney studies. In the SUSTAIN 1–7 post hoc kidney analysis, semaglutide was associated with initial eGFR reductions that subsequently plateaued, with marked uACR reductions and no kidney safety signal versus comparators [16]. The dedicated FLOW trial demonstrated hard kidney outcome benefit and a slower annual eGFR slope favouring semaglutide, without any absolute eGFR increase [19]. Thus, the short-term eGFR rise observed here should not be interpreted as structural improvement but rather as a measurement phenomenon requiring longer follow-up and comparator-controlled designs to distinguish from true disease-modifying change [16,19].

The central and most clinically credible renal finding is the 19.8% reduction in geometric mean uACR (GMR 0.80; ln[uACR] mean change −0.22, 95% CI: −0.28 to −0.16; *p* < 0.001). This magnitude is highly concordant with the GLP-1RA trial literature, where uACR reductions of 20–40% have been consistently reported, particularly in patients with baseline albuminuria [13,16,24]. The SUSTAIN 1–7 post hoc analysis demonstrated marked uACR reductions with greater benefit in those with higher baseline albuminuria [16]. Exploratory STEP trial analyses confirmed albuminuria-lowering in populations with overweight or obesity, extending the signal beyond a diabetes-only context [17]. The FLOW trial confirmed that semaglutide-mediated albuminuria reduction translates into a reduction in hard kidney outcomes, strengthening the interpretation that short-term improvements in uACR may represent an intermediate phenotype aligned with longer-term kidney protection [19].

G-category improvement occurred in 17.3%, stability in 76.8%, and worsening in 5.9%. For albuminuria categories, 95.7% remained stable, 3.1% improved, and 1.2% worsened. Category movement is threshold-dependent and sensitive to short-term biological variability, particularly when values lie near category cutoffs [21]. The majority of GFR category improvements were G2 → G1 crossings explained by the creatinine artefact described above. The modest category transition proportions should therefore be interpreted as supportive but not definitive evidence of CKD stage reversal, consistent with the need for repeated confirmed measurements before ascribing categorical change [21].

Using clinically interpretable thresholds, 14.8% achieved ≥30% uACR reduction overall, rising to 66.7% among those with baseline uACR ≥ 30 mg/mmol (A3). This pattern matches the baseline-response gradient: proportional albuminuria reductions are largest when baseline albuminuria is highest, reflecting both greater absolute room for improvement and a phenotype more responsive to therapies that reduce intraglomerular pressure and inflammation [16,24]. These responder proportions complement group-level biomarker changes by quantifying clinically meaningful individual responses.

### 4.4. Subgroup, Correlation, and Sensitivity Analyses

Participants with baseline eGFR < 60 mL/min/1.73 m^2^ (n = 56) showed larger improvements in eGFR (6.7 mL/min/1.73 m^2^) and creatinine (−11.6 µmol/L), with a significant ln(uACR) reduction (−0.35). Those with baseline uACR ≥ 30 mg/mmol (n = 15) showed a marked albuminuria signal (Δln[uACR] −1.41). While directionally consistent with risk-enriched populations benefiting more in GLP-1RA trial analyses [16,19], these findings should be treated as exploratory given subgroup sizes and the well-recognised susceptibility to regression to the mean. The inverse association between higher baseline uACR and greater ln(uACR) reduction (ρ −0.540) is consistent with trial analyses showing greater albuminuria-lowering in those with baseline micro- or macroalbuminuria [16,24]. The inverse correlation between lower baseline eGFR and larger eGFR increase (ρ −0.355) is more likely to reflect creatinine artefacts and regression to the mean than genuine disproportionate filtration benefit.

Sensitivity analyses (n = 296) demonstrated consistent direction and statistical significance for BMI, creatinine, eGFR, and ln(uACR) (all *p* < 0.001), supporting robustness of the primary conclusions. Such consistency strengthens confidence that the principal findings are not driven solely by extreme values or influential observations, though it does not replace the need for a controlled comparator to support causal inference.

### 4.5. Integration with the Evidence Base, Mechanisms, and Clinical Implications

The GLP-1RA kidney evidence base has evolved from signal generation in cardiovascular outcomes trials to definitive proof in a dedicated kidney outcomes trial. Earlier trials—the SUSTAIN-6 semaglutide and LEADER liraglutide renal outcome analyses—demonstrated kidney benefit through albuminuria-based composite endpoints [15,26]. The AWARD-7 trial specifically demonstrated eGFR slope preservation and albuminuria reduction with dulaglutide versus insulin glargine in patients with T2D and moderate-to-severe CKD, establishing proof-of-concept for GLP-1RA kidney protection in a CKD-enriched population prior to FLOW [27]. Subsequent meta-analyses confirmed class-wide consistency in kidney endpoint reduction [13,14,24]. The FLOW trial closed the evidentiary gap by demonstrating hard kidney outcome reduction in a CKD-enriched T2D population [19]. The SELECT kidney analysis extended semaglutide’s renal benefits to patients without T2D, reinforcing the plausibility of weight-mediated kidney mechanisms [18]. Of additional clinical relevance, the FIDELIO-DKD trial demonstrated that finerenone, a non-steroidal mineralocorticoid receptor antagonist, provides kidney protection on top of RAAS blockade in T2D and CKD, underscoring the substantial residual risk that persists despite foundational therapy and the value of combinatorial cardio-renal strategies of which GLP-1RAs are a part [28].

GLP-1RA-mediated kidney protection is multi-mechanistic, encompassing indirect and potentially direct renal pathways. The pathophysiology of DKD itself involves the convergence of haemodynamic, metabolic, and inflammatory mechanisms that collectively drive progressive nephron loss [29]. Indirect GLP-1RA mechanisms include weight reduction and attendant improvements in intraglomerular haemodynamics, glycaemic improvement and attenuation of glucotoxic injury, and reductions in systemic inflammation and endothelial dysfunction. Putative direct renal mechanisms include natriuresis and tubular effects, and anti-inflammatory and anti-oxidative pathways supported by preclinical and translational evidence, including inhibition of NF-κB-mediated inflammatory signalling in the proximal tubule [24,30]. Recent high-level reviews emphasise that reduced kidney inflammation is a leading candidate direct mechanism, although definitive human phenotyping remains incompletely established [24].

KDIGO guidance for diabetes management in CKD supports use of GLP-1RAs as part of a layered approach to risk reduction, particularly for patients needing additional glycaemic control and weight reduction beyond foundational kidney-protective therapies [20]. The present findings support the pragmatic clinical expectation that semaglutide initiation can yield meaningful short-term improvements in weight, glycaemic control, and albuminuria in routine care, with kidney function biomarkers trending favourably over six months when interpreted with appropriate caveats.

### 4.6. Strengths and Limitations

Several methodological strengths merit acknowledgement. This study represents, to our knowledge, the first real-world paired cohort analysis of semaglutide kidney and metabolic biomarker outcomes from the MENA region, addressing a well-documented geographic gap in the global real-world evidence base. The KDIGO Cause–GFR–Albuminuria framework was systematically applied using SI units, enabling KDIGO-aligned category transition reporting that is directly interpretable for practising nephrologists, an approach not consistently adopted in existing observational studies. Pre-specified subgroup and sensitivity analyses were conducted a priori, with all exploratory findings explicitly labelled throughout.

Notwithstanding these strengths, several limitations must be acknowledged. The single-arm pre–post design, without an untreated or active comparator, precludes causal attribution of the observed changes to semaglutide. Concurrent care changes, including medication adjustments, dietary modification, and spontaneous biomarker fluctuation, represent uncontrolled confounders. The six-month follow-up is insufficient to characterise CKD progression, durability of albuminuria benefit, or kidney failure endpoints, all of which require multi-year observation. Foundational therapies were generally stable over the six-month follow-up period, with no major changes in RAAS blockade, SGLT2 inhibitors, or mineralocorticoid receptor antagonists identified. Granular longitudinal adjudication of individual medication changes was not performed. The possibility that incidental alterations in background therapy contributed independently to the observed biomarker changes cannot be excluded and is an inherent limitation of this retrospective design.

Single-time-point creatinine, eGFR, and uACR measurements are subject to biological and assay variability. CKD chronicity was confirmed from established clinical diagnoses and available biochemical records; prospective repeated confirmed measurements were not available for every participant, which is an inherent constraint of the retrospective design. The external validity of these findings is also constrained by the study population and setting. The cohort was drawn from a single secondary-care hospital in the United Arab Emirates, was predominantly female, and comprised mainly early-stage, albuminuria-driven disease. The findings therefore apply principally to this phenotype and may not generalise to male-predominant populations, to those with advanced GFR impairment or heavy albuminuria, or to other care settings and health systems. Furthermore, the cohort comprised predominantly mild CKD, with most participants having preserved eGFR (KDIGO G1–G2) and moderately increased albuminuria (category A2), and only a small number with more severe kidney disease. The number of participants with advanced eGFR impairment or heavy albuminuria was insufficient to permit any reliable assessment of semaglutide-associated biomarker responses in these more severe categories, and the findings should not be extrapolated to them. Confirmation in multicentre cohorts across diverse populations is needed.

## 5. Conclusions

In a real-world cohort of adults with T2D and mild CKD characterised by substantial obesity and predominantly early-stage albuminuric disease, six months of semaglutide therapy was associated with clinically meaningful improvements in body weight, glycaemic control, and albuminuria. The reduction in geometric mean uACR is concordant with reductions consistently reported across the GLP-1RA trial evidence base. The continuous inverse relationship between baseline albuminuria and treatment response further supports the biological plausibility of semaglutide-mediated kidney protection in routine clinical practice. The parallel reduction in serum creatinine and rise in eGFR are attributable in large part to weight-related reductions in creatinine generation rather than structural filtration improvement, and should be interpreted accordingly. Taken together, these findings confirm that trial-level metabolic and renal biomarker expectations translate to a real-world secondary-care setting. They support semaglutide as a clinically meaningful component of layered cardio-renal protective therapy alongside RAAS blockade and SGLT2 inhibitors in patients with T2D and mild CKD. For the practising clinician, these data indicate that the metabolic and albuminuria benefits observed in controlled trials can be expected to translate to routine secondary-care management. These are hypothesis-generating data from the first KDIGO-aligned paired cohort study in the MENA region. They contribute geographic and methodological breadth to the post-FLOW real-world evidence landscape, and highlight the need for prospective, comparator-controlled studies that confirm the durability of the albuminuria benefit, evaluate hard kidney endpoints over longer follow-up, and extend these observations to multicentre cohorts across diverse populations.

## Figures and Tables

**Table 1:** Baseline characteristics of the study population.

Characteristic	Value
Participants, n	324
Age, years	55.3 ± 12.4
Female sex, n (%)	214/324 (66.0)
BMI, kg/m^2^	36.1 ± 6.9
HbA1c, %	8.3 [7.2, 10.0] (n = 298)
Serum creatinine, µmol/L	80.5 ± 36.3
eGFR, mL/min/1.73 m^2^	87.0 ± 25.8 (n = 323)
uACR, mg/mmol	7.7 [6.5, 9.9]
uACR geometric mean, mg/mmol	8.8
CKD Stage (KDIGO G category), n (%)	
G1 (≥90)	176/323 (54.5)
G2 (60–89)	91/323 (28.2)
G3a (45–59)	30/323 (9.3)
G3b (30–44)	17/323 (5.3)
G4 (15–29)	9/323 (2.8)
CKD Stage (KDIGO A category), n (%)	
A1 (<3) mg/mmol	9/324 (2.8)
A2 (3–30) mg/mmol	300/324 (92.6)
A3 (>30) mg/mmol	15/324 (4.6)
Background Pharmacotherapy	
RAAS blockade (ACEi or ARB), n (%)	231/324 (71.4)
SGLT2 inhibitor, n (%)	227/324 (70.1)
Non-steroidal MRA (finerenone), n (%)	15/324 (4.6)
Insulin, n (%)	32/324 (10.0)
Sulfonylurea (gliclazide), n (%)	49/324 (15.1)

Data are presented as mean ± SD, median [IQR], or n (%), as appropriate. ACEi: angiotensin-converting enzyme inhibitor; ARB: angiotensin receptor blocker; MRA: mineralocorticoid receptor antagonist; SGLT2: sodium–glucose cotransporter-2; RAAS: renin–angiotensin–aldosterone system.

**Table 2:** Within-participant changes in metabolic and kidney biomarkers from baseline to 6 months.

Outcome	Paired n	Baseline	6 Months	Mean Change (95% CI)	Median Change [IQR]	*p* Value
BMI (kg/m^2^)	323	36.1 ± 6.9	34.1 ± 6.8	−2.0 (−2.2 to −1.8)	−2.0 [−3.0, −1.0]	<0.001 *
HbA1c (%)	298	8.5 ± 2.0	6.2 ± 1.4	−2.4 (−2.6 to −2.2)	−2.0 [−3.3, −1.1]	<0.001 ^#^
Serum creatinine (µmol/L)	324	80.5 ± 36.3	76.9 ± 32.7	−3.6 (−5.4 to −1.8)	−3.0 [−10.0, 3.0]	<0.001 *
eGFR (mL/min/1.73 m^2^)	323	87.0 ± 25.8	90.7 ± 24.7	3.7 (2.4 to 5.0)	3.0 [−2.0, 10.0]	<0.001 *
ln(uACR) [mg/mmol]	324	2.19 ± 0.79	1.97 ± 0.60	−0.22 (−0.28 to −0.16)	−0.12 [−0.25, −0.05]	<0.001 ^#^

Data are presented as mean ± SD, median [IQR], or n (%), as appropriate. * = paired *t*-test, ^#^ = Wilcoxon signed-rank test. uACR is reported in mg/mmol; KDIGO albuminuria categories for mg/mmol are A1 < 3, A2 3–30, and A3 > 30 mg/mmol. uACR was analysed on the natural log scale; the geometric mean ratio is exp(mean change in ln[uACR]). *p* values are two-sided.

**Table 3:** KDIGO GFR and albuminuria category shifts from baseline to 6 months.

Endpoint	Value
KDIGO G category shift (baseline → 6 months)	
Improved	56/323 (17.3%)
Stable	248/323 (76.8%)
Worsened	19/323 (5.9%)
KDIGO A category shift (baseline → 6 months) [mg/mmol]	
Improved	10/324 (3.1%)
Stable	310/324 (95.7%)
Worsened	4/324 (1.2%)

**Table 4:** Clinically interpretable endpoint proportions at 6 months.

Responder Endpoint	Value
BMI reduction ≥ 5% from baseline	168/323 (52.0%)
HbA1c reduction ≥ 0.5% absolute	260/298 (87.2%)
eGFR decline ≥ 30% from baseline	3/323 (0.9%)
uACR reduction ≥ 30% from baseline (mg/mmol)	48/324 (14.8%)
uACR reduction ≥ 30% among baseline uACR ≥ 30 mg/mmol (KDIGO A3)	10/15 (66.7%)

## Data Availability

The original contributions presented in this study are included in the article/Supplementary Materials. Further inquiries can be directed to the corresponding author(s).

## Supplementary Materials

The following supporting information can be downloaded at https://www.mdpi.com/article/10.3390/jcm15145577/s1: Table S1. Sensitivity analyses: within-participant biomarker changes from baseline to 6 months. Table S2. Subgroup analyses of kidney outcomes at 6 months by baseline kidney risk strata. File S1. STROBE Statement—Checklist of Items for Reporting Observational Studies.

## List of Abbreviations

ACEi	Angiotensin-converting enzyme inhibitor
ARB	Angiotensin receptor blocker
BMI	Body mass index
CGA	Cause–GFR–Albuminuria (KDIGO risk framework)
CI	Confidence interval
CKD	Chronic kidney disease
CKD-EPI	Chronic Kidney Disease Epidemiology Collaboration
DKD	Diabetic kidney disease
eGFR	Estimated glomerular filtration rate
ESKD	End-stage kidney disease
GFR	Glomerular filtration rate
GLP-1RA	Glucagon-like peptide-1 receptor agonist
GMR	Geometric mean ratio
HbA1c	Glycated haemoglobin
IQR	Interquartile range
KDIGO	Kidney Disease: Improving Global Outcomes
MACE	Major adverse cardiovascular events
MENA	Middle East and North Africa
MRA	Mineralocorticoid receptor antagonist
RAAS	Renin–angiotensin–aldosterone system
SGLT2i	Sodium–glucose cotransporter-2 inhibitor
SD	Standard deviation
STROBE	Strengthening the Reporting of Observational Studies in Epidemiology
T2D	Type 2 diabetes mellitus
uACR	Urinary albumin-to-creatinine ratio
UAE	United Arab Emirates
