# Supplementary material for: Real-World Kidney and Glycaemic Outcomes Following Semaglutide Initiation in Adults with Type 2 Diabetes and Mild Chronic Kidney Disease
Source: J Clin Med. 2026 Jul 16;15(14):5577. doi: 10.3390/jcm15145577 (PMC13412203; doi:10.3390/jcm15145577)
Supplement: Supplementary file 1 [file jcm-15-05577-s001.zip › jcm-4312473-supplementary.pdf]

Table S1. Sensitivity analyses: within-participant biomarker changes from baseline to 6 months

Outcome	n	Baseline	6 months	Mean change (95% CI)	Median change [IQR]	P value
BMI, kg/m^2	296	36.2 ± 7.0	34.2 ± 6.9	-1.97 (-2.21 to -1.73)	-2.0 [-3.0, -1.0]	<0.001*
HbA1c, %	296	8.5 ± 2.0	6.1 ± 1.4	-2.4 (-2.6 to -2.2)	-2.0 [-3.3, -1.1]	<0.001#
Serum creatinine, µmol/L	296	80.4 ± 36.2	76.7 ± 32.4	-3.68 (-5.69 to -1.68)	-3.0 [-10.0, 3.0]	<0.001*
eGFR, mL/min/1.73 m^2	296	87.1 ± 25.7	91.0 ± 24.9	+3.89 (+2.51 to +5.27)	+3.0 [-2.0, 10.0]	<0.001*
ln(uACR), mg/mmol	296	2.18 ± 0.66	1.97 ± 0.57	-0.21 (-0.28 to -0.15)	-0.12 [-0.26, -0.05]	<0.001#

* Paired t-test, # Wilcoxon signed-rank

Table S2. Subgroup analyses of kidney outcomes at 6 months by baseline kidney risk strata

Subgroup / Outcome	Paired n	Effect estimate	P value
Baseline eGFR <60 mL/min/1.73 m^2 (n=56)			
Δ eGFR (mL/min/1.73 m^2)	56	+6.7 (3.2 to 10.2)	<0.001
Δ Creatinine (µmol/L)	56	-11.6 (-19.2 to -4.0)	0.003
Δ ln(uACR) [mg/mmol]	56	-0.35 (-0.59 to -0.12)	<0.001
Baseline uACR ≥30 mg/mmol (KDIGO A3) (n=15)			
Δ ln(uACR) [mg/mmol]	15	-1.41 (-2.28 to -0.54)	<0.001

Supplementary File S1

STROBE Statement — Checklist of Items for Reporting Observational Studies

Section / Topic	No.	Recommendation	Page No.	Relevant text from manuscript
**Title and Abstract**				
**Title and abstract**	1a	(a) Indicate the study's design with a commonly used term in the title or the abstract	p. 1	Title: 'Real-World Kidney and Metabolic Outcomes Following Semaglutide Initiation in Adults with Type 2 Diabetes and Chronic Kidney Disease.' Abstract Methods: 'We conducted a retrospective, single-centre, paired-cohort study...'
	1b	(b) Provide in the abstract an informative and balanced summary of what was done and what was found	p. 1	Structured abstract with Background, Methods, Results, Conclusions. Key quantitative findings reported (BMI -2.0 kg/m², HbA1c -2.0%, uACR -19.8%, KDIGO category transitions). Single-arm caveat: 'findings describe biomarker trajectories and cannot establish causality.'
**Introduction**				
**Background/rationale**	2	Explain the scientific background and rationale for the investigation being reported	pp. 1-3	Introduction (Section 1) covers: global DKD burden; residual risk despite RAAS blockade and SGLT2 inhibitors; emerging GLP-1RA kidney evidence culminating in FLOW; MENA underrepresentation in real-world evidence; KDIGO CGA methodological gap.
**Objectives**	3	State specific objectives, including any prespecified hypotheses	p. 3	Final paragraph of Introduction states the central research question and the primary objective (within-participant six-month changes in serum creatinine, eGFR, uACR) and secondary objectives (BMI, HbA1c, KDIGO G and A category transitions, pre-specified responder proportions). Study explicitly framed as hypothesis-generating rather than a causal efficacy test.
**Methods**				
**Study design**	4	Present key elements of study design early in the paper	p. 3	Methods first sentence: 'This was a retrospective, single-center, observational cohort study conducted at Ibrahim Bin Hamad Obaidallah Hospital, Ras Al

				Khaimah, United Arab Emirates.' Single-arm pre-post paired design without comparator.
**Setting**	5	Describe the setting, locations, and relevant dates, including periods of recruitment, exposure, follow-up, and data collection	p. 3	Setting: Ibrahim Bin Hamad Obaidallah Hospital, Ras Al Khaimah, UAE (secondary care). Recruitment/initiation: June-December 2024. Follow-up: 6 months (5-7 months post-initiation). Data extraction: June 2025. Ethics: MOHAP/REC/2025/22-2025-UG-P.
**Participants**	6a	Cohort study - Give the eligibility criteria, and the sources and methods of selection of participants. Describe methods of follow-up	pp. 3-4	Source: electronic medical records. Inclusion: age >=18; T2DM + CKD (KDIGO 2022: uACR >=3 mg/mmol and/or eGFR <90 mL/min/1.73 m², >=3 months); newly initiated semaglutide June-December 2024; baseline within 4 weeks; >=1 paired 6-month outcome. Exclusion: eGFR <15 or RRT; prior GLP-1RA within 12 months; SGLT2i within 3 months; active malignancy; pregnancy. Chronicity confirmed from clinical diagnosis or >=2 measurements >=3 months apart.
	6b	Cohort study - For matched studies, give matching criteria and number of exposed and unexposed	N/A	Not applicable. This is not a matched cohort study.
**Variables**	7	Clearly define all outcomes, exposures, predictors, potential confounders, and effect modifiers. Give diagnostic criteria, if applicable	pp. 3-4	Exposure: semaglutide 0.25 mg to 0.5 mg to 1.0 mg (standard escalation). Primary: change in creatinine, eGFR (CKD-EPI 2021), ln(uACR). Secondary: change in BMI, HbA1c, KDIGO G/A shifts. Thresholds: BMI >=5%, HbA1c >=0.5%, eGFR >=30% decline, uACR >=30%. Background therapy documented in Table 1 (p. 5).
**Data sources/measurement**	8	For each variable of interest, give sources of data and details of methods of assessment (measurement)	pp. 3-4	Data: electronic medical records. eGFR: CKD-EPI 2021 (race-free). uACR: mg/mmol from routine laboratory. Baseline: within 4 weeks of initiation. Follow-up: 5-7 months post-initiation. Blood pressure and lipid parameters not systematically available and therefore not assessed (stated explicitly p. 4).
**Bias**	9	Describe any efforts to address potential sources of bias	pp. 3-4, 8-9, 11-12	Single-arm limitation stated throughout. Sensitivity analysis (n=296) for missing-data bias. Creatinine generation artefact discussed (Section 4.3). Regression-to-mean addressed for subgroup analyses (Section 4.4). Medication stability confirmed: 'foundational therapies were generally

				stable' (p. 3-4). CKD chronicity confirmation described (p. 3). Participant flow with exclusions shown in Figure 1.
**Study size**	10	Explain how the study size was arrived at	pp. 3-5 Figure 1	No a priori sample-size calculation; all consecutively eligible patients initiated on semaglutide during the study period were included, representing a census of the available population (stated in Methods). Total screened: n=528; excluded: n=204; final cohort: n=324 (Figure 1).
**Quantitative variables**	11	Explain how quantitative variables were handled in the analyses. If applicable, describe which groupings were chosen and why	p. 4	uACR analysed on natural log scale (right-skewed); GMR = exp(mean change in ln[uACR]). KDIGO GFR thresholds: G1 >=90, G2 60-89, G3a 45-59, G3b 30-44, G4 15-29 mL/min/1.73 m². KDIGO A thresholds: A1 <3, A2 3-30, A3 >30 mg/mmol. Normally distributed outcomes: mean ± SD; non-normal: median [IQR].
**Statistical methods**	12a	(a) Describe all statistical methods, including those used to control for confounding	p. 4	IBM SPSS v29. Normality: Shapiro-Wilk. Paired t-test (BMI, creatinine, eGFR); Wilcoxon signed-rank (HbA1c, ln[uACR]). No confounder adjustment (single-arm pre-post design). All tests two-sided; p<0.05 significant.
	12b	(b) Describe any methods used to examine subgroups and interactions	p. 4	Pre-specified subgroups: baseline eGFR <60 mL/min/1.73 m² (n=56); baseline uACR >=30 mg/mmol (KDIGO A3; n=15). Exploratory Spearman rank correlations: baseline uACR vs change in ln(uACR); baseline eGFR vs change in eGFR. Subgroup and correlation analyses stated to be exploratory and hypothesis-generating.
	12c	(c) Explain how missing data were addressed	p. 4	Complete-case approach per outcome. Of 26 without paired HbA1c: 23 missing baseline, 3 missing follow-up. Comparison of HbA1c-missing vs complete baseline characteristics performed and reported.
	12d	(d) Cohort study - If applicable, explain how loss to follow-up was addressed	pp. 3-4	Initiation window closed December 2024; data extracted June 2025 ensuring minimum 6-month follow-up. Patients with >=1 paired 6-month outcome eligible. Varying n per outcome reflects available complete paired data.

	12e	(e) Describe any sensitivity analyses	pp. 4, 7<br>Table S1	Sensitivity analysis: participants with complete paired data across all outcomes (n=296). Results consistent in direction and magnitude for all outcomes (all p<0.001), Table S1.
**Results**				
**Participants**	13a	(a) Report numbers of individuals at each stage of study	pp. 4-5<br>Figure 1	Screened: 528. Excluded: 204. Included: 324. Paired data: creatinine/uACR n=324; eGFR/BMI n=323; HbA1c n=298. Complete all outcomes (sensitivity): n=296. Participant flow diagram presented as Figure 1 in the main text.
	13b	(b) Give reasons for non-participation at each stage	pp. 3-4<br>Figure 1	Exclusion reasons (Methods; Figure 1): did not meet KDIGO CKD criteria; eGFR <15 or RRT; prior GLP-1RA within 12 months; SGLT2i within 3 months; active malignancy; pregnancy; insufficient paired data. Individual per-criterion counts not recorded (retrospective design limitation).
	13c	(c) Consider use of a flow diagram	Figure 1<br>(main text)	STROBE-compliant participant flow diagram presented as Figure 1 in the main text, showing records identified (n=528), exclusions with reasons (n=204), final cohort (n=324), and analytical sample sizes per outcome.
**Descriptive data**	14a	(a) Give characteristics of study participants	pp. 4-5<br>Table 1	Table 1: age 55.3±12.4 years, 66.0% female, BMI 36.1±6.9 kg/m², HbA1c 8.3 [7.2-10.0]%, creatinine 80.5±36.3 μmol/L, eGFR 87.0±25.8 mL/min/1.73 m², uACR 7.7 [6.5-9.9] mg/mmol, KDIGO G/A categories, background pharmacotherapy (RAAS 71.4%, SGLT2i 70.1%, MRA 4.6%, insulin 10.0%, sulfonylurea 15.1%).
	14b	(b) Indicate number of participants with missing data for each variable of interest	pp. 4-5	HbA1c: 26/324 missing (23 baseline, 3 follow-up). eGFR and BMI: 1 missing each (n=323). Creatinine and uACR: complete (n=324). Stated in Results Section 3.1 and Methods.
	14c	(c) Cohort study - Summarise follow-up time	p. 4	Uniform 6-month follow-up (5-7 months post-initiation), stated in Methods.
**Outcome data**	15	Cohort study - Report numbers of outcome events or summary measures over time	pp. 5-7<br>Tables 2-4	Table 2: within-participant changes with 95% CI at 6 months for all five outcomes. Table 3: KDIGO G/A category transitions (improved/stable/worsened). Table 4: clinically interpretable responder

				proportions. GMR 0.80 representing 19.8% uACR reduction.
**Main results**	16a	(a) Give unadjusted estimates and, if applicable, confounder-adjusted estimates and their precision (eg, 95% CI)	pp. 5-7 Tables 2-4	All outcomes reported as mean change (95% CI) or median change [IQR] with p-values. No confounder adjustment (pre-post paired design). Wilson score 95% CI for responder proportions. Tables 2 and 4; Figure 3.
	16b	(b) Report category boundaries when continuous variables were categorized	pp. 4, 6 Table 3	KDIGO GFR: G1 >=90, G2 60-89, G3a 45-59, G3b 30-44, G4 15-29 mL/min/1.73 m². KDIGO A: A1 <3, A2 3-30, A3 >30 mg/mmol. Defined in Methods (p. 4) and reported in Table 3 and Section 3.4.
	16c	(c) If relevant, consider translating estimates of relative risk into absolute risk for a meaningful time period	pp. 6-7 Table 4	Absolute responder proportions: BMI >=5% reduction 168/323 (52.0%); HbA1c >=0.5% 260/298 (87.2%); eGFR >=30% decline 3/323 (0.9%); uACR >=30% reduction 48/324 (14.8%) overall, 10/15 (66.7%) in KDIGO A3, with 95% Wilson CI (Table 4; Figure 3).
**Other analyses**	17	Report other analyses done - eg analyses of subgroups and interactions, and sensitivity analyses	p. 7 Tables S1-S2	Sensitivity analysis n=296 (Table S1). Pre-specified subgroups: eGFR <60 n=56; KDIGO A3 n=15 (Table S2). Exploratory Spearman: baseline uACR vs change in ln(uACR) rho=-0.540 (p<0.001); baseline eGFR vs change in eGFR rho=-0.355 (p<0.001). All subgroup findings labelled exploratory and hypothesis-generating (Section 3.6).
**Discussion**				
**Key results**	18	Summarise key results with reference to study objectives	pp. 7-11	Discussion (Sections 4.1-4.5) summarises findings against objectives: baseline phenotype (4.1); metabolic effects on weight and glycaemic control (4.2); kidney biomarker effects including the creatinine artefact and albuminuria as primary credible finding (4.3); subgroup, correlation, and sensitivity analyses (4.4); integration with the GLP-1RA evidence base, mechanisms, and clinical implications (4.5).
**Limitations**	19	Discuss limitations of the study, taking into account sources of potential bias or imprecision	pp. 11-12	Section 4.6 (Strengths and Limitations): single-arm design and no causal attribution; six-month follow-up insufficient for hard outcomes; creatinine artefact; granular medication data not adjudicated; single time-point measurement variability; CKD chronicity

				confirmation; external validity constrained by single-centre UAE setting, female predominance, and early-stage albuminuria-driven phenotype.
**Interpretation**	20	Give a cautious overall interpretation of results considering objectives, limitations, multiplicity of analyses, results from similar studies, and other relevant evidence	pp. 10-12	Section 4.5 contextualises findings against FLOW, SUSTAIN 1-7, LEADER, AWARD-7, SELECT, FIDELIO-DKD, and meta-analyses. eGFR rise attributed to creatinine artefact. Subgroups labelled exploratory. Conclusions maintain hypothesis-generating framing and identify required future studies.
**Generalisability**	21	Discuss the generalisability (external validity) of the study results	pp. 7-8, 11-12	Section 4.1: findings apply to early-stage albuminuria-driven CKD; not to be extrapolated to KDIGO G4-G5 or ESKD. Section 4.6: single-centre UAE setting, female predominance, and early-stage phenotype constrain external validity; confirmation in multicentre cohorts across diverse populations identified as needed.
**Other Information**				
**Funding**	22	Give the source of funding and the role of the funders for the present study	p. 11	Funding statement present: 'No funding was received for this work.'
